# Sensor-Based Detection of Predator Influence on Livestock: A Case Study Exploring the Impacts of Wild Dogs (*Canis familiaris*) on Rangeland Sheep

**DOI:** 10.3390/ani12030219

**Published:** 2022-01-18

**Authors:** Caitlin A. Evans, Mark G. Trotter, Jaime K. Manning

**Affiliations:** Institute for Future Farming Systems, School of Health, Medical and Applied Sciences, CQUniversity Australia, Rockhampton, QLD 4701, Australia; m.trotter@cqu.edu.au (M.G.T.); j.k.manning@cqu.edu.au (J.K.M.)

**Keywords:** behaviour, on-animal sensors, predation, rangelands, sheep

## Abstract

**Simple Summary:**

Sheep predation by wild dogs has serious production and animal welfare implications. By monitoring changes in the behaviour of sheep, on-animal sensors are an option for detecting wild dogs and alerting producers to their presence. This study identified differences in the daily distance travelled of sheep when in the presence and absence of a wild dog and highlights the potential for on-animal sensors to be used as a monitoring and management tool for wild dog detection.

**Abstract:**

In Australia, wild dogs are one of the leading causes of sheep losses. A major problem with managing wild dogs in Australia’s rangeland environments is that sheep producers are often unaware of their presence until injuries or deaths are observed. One option for earlier detection of wild dogs is on-animal sensors, such as Global Positioning System (GPS) tracking collars, to detect changes in the behaviour of sheep due to the presence of wild dogs. The current study used spatio-temporal data, derived from GPS tracking collars, deployed on sheep from a single rangeland property to determine if there were differences in the behaviour of sheep when in the presence, or absence, of a wild dog. Results indicated that the presence of a wild dog influenced the daily behaviours of sheep by increasing the daily distance travelled. Differences in sheep diurnal activity were also observed during periods where a wild dog was present or absent on the property. These results highlight the potential for on-animal sensors to be used as a monitoring tool for sheep flocks directly impacted by wild dogs, although further work is needed to determine the applicability of these results to other sheep production regions of Australia.

## 1. Introduction

Australian sheep producers have struggled with stock loss due to predation from as early on as the 1890s [1]. While the predation of sheep and lambs can be due to a range of species, including foxes and wild pigs, wild dogs are a particular problem for producers and have been reported as one of the major causes of declining sheep numbers in Australia [2,3]. In Australia, the term wild dog refers to all members of *Canis familiaris,* such as dingoes, feral domestic dogs and their hybrids [4,5]. Wild dogs can be found over much of mainland Australia; however, their abundance is higher in northern pastoral regions due to the National wild dog barrier fence that spans over 5400 km from South Australia to eastern Queensland, with an aim to protect southern pastoral areas from high wild dog numbers [6]. Wild dog predation has been estimated to cost the Australian lamb and wool industries between AUD 21.85 million [7] and AUD 26.78 million per year [8], and the increasing losses associated with wild dogs have forced many producers to leave these industries in regions prone to high wild dog numbers [9]. In addition to the economic and production impacts of wild dog predation, there are severe animal welfare concerns, with the likelihood of injuries sustained during these attacks remaining undetected by producers for extended periods due to the extensive nature of sheep properties in Australia and infrequent monitoring [10,11]. Finally, the severity and frequency of wild dog attacks in some areas can take an emotional toll on producers and lead to social implications, such as psychological distress and financial stress [12] that are often overlooked [13].

Wild dogs tend to have set home ranges, although they can travel between 9 and 21 km/day and have been found up to 1300 km from their point of origin [14]. The capacity of wild dogs to persist in diverse environments, the extensive distances they can travel and the production and animal welfare implications of attacks by wild dogs on sheep highlights the need for control options for sheep producers. In Australia, options for wild dog control are varied and include lethal (i.e., targeted baiting, shooting and trapping programs) and non-lethal methods (i.e., National wild dog barrier fence or livestock guardian animals) [9,15,16]. While both lethal and non-lethal control options have shown to be effective at reducing wild dog numbers, or the number of livestock lost to wild dogs [15,16], these control methods are not sufficient to reduce the impacts from wild dogs to a level that enables viable sheep production in many areas north of the National wild dog barrier fence [9].

A major problem with managing wild dog populations in rangeland environments is that producers are often unaware of their presence in the area until injuries or deaths are observed. As such, the development of additional methods for identifying and managing wild dogs to work along with current control methods is necessary [17]. One option for identifying the presence of wild dogs is the use of remote livestock monitoring technologies, such as on-animal sensors, which have the potential to improve the monitoring and welfare of sheep [18] and detect changes in sheep behaviour [19]. The value of on-animal sensors in detecting predation and reducing sheep losses has been estimated to save AUD 80 million over 30 years for the Australian sheep sector [20], with producers also realising the potential of on-animal sensors as an option in reducing the economic losses associated with predation [21]. Recently, Manning et al. [22] reported that on-animal sensors were able to quantify the behavioural responses of sheep during a simulated dog predation event by identifying increases in the velocity and centripetal rotation (behaviour) of animals when approached by dogs. However, it is not well understood whether there are other generalised movements or behavioural changes that sheep exhibit in the presence of wild dogs under commercial, rangeland conditions and how these changes could assist in earlier identification and/or confirmation of wild dog activity in an area.

In this study, GPS tracking collars were deployed on a single large-scale sheep grazing property in August 2019, as part of a larger project looking at the suitability of on-animal sensors for rangeland sheep. During this time, a wild dog was active on the property, indicated by signs (tracks/scats) and sightings of the wild dog in close proximity to sheep. Subsequently, the wild dog was shot, and no other wild dogs were identified on the property during the study period. This series of events, whilst unplanned, provided an excellent opportunity to explore the differences in the behaviour of sheep both initially while the dog was present and subsequently after the dog was removed. As such, the aim of this paper was to determine if spatio-temporal data derived from on-animal GPS tracking collars could identify differences in the behaviour of extensively grazed sheep when in the presence, or absence, of a wild dog. It was hypothesised that sheep would increase their daily activity levels when in the presence of a wild dog.

## 2. Materials and Methods

### 2.1. Location and Animals

**Figure 1:**
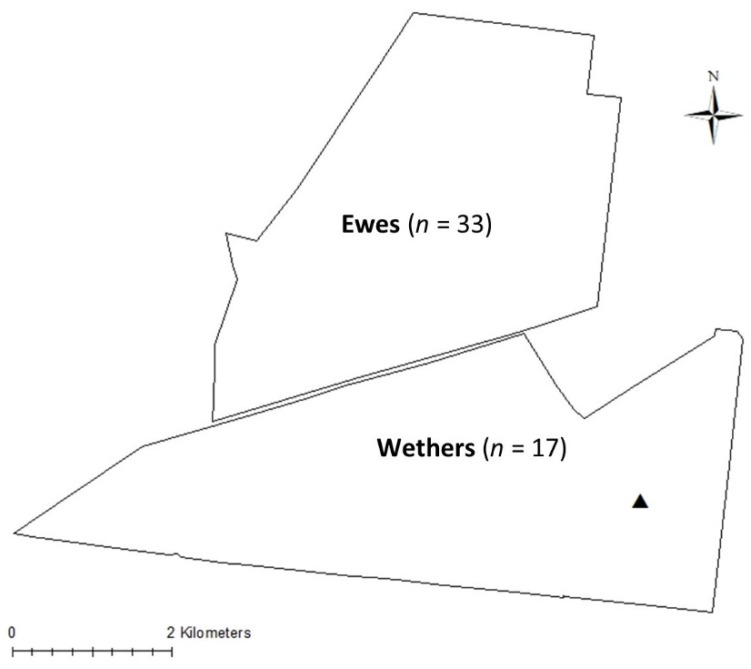
Map of study paddocks and location of wild dog den (▲). Paddock sizes were 1717 ha and 2009 ha for ewes and wethers, respectively. Sample sizes of ewe and wether groups refer to the final number of GPS tracking collars analysed.

The broader study, looking at the suitability of on-animal sensors for rangeland sheep, was approved by the CQUniversity Australia animal ethics committee (ethics approval number 21540). Data were obtained from a commercial sheep property in Western Queensland, Australia. The 25,900-hectare property is situated approximately 20 km southwest of Barcaldine. For the current study, two paddocks of approximately 1700 and 2000 hectares in size (Figure 1) were used with water sources consisting of water impoundment dams and/or water troughs.

Wild dogs had been a long-term problem for the study property, which had resulted in the erection of a wild dog exclusion fence around the boundary of the property. In the preceding month leading up to the study, three wild dogs had been identified and shot on sight, with a single wild dog identified by a licensed dog trapper during the study period. Signs of the wild dog were observed by way of fresh tracks/scats or visual observations on the 12th, 15th, 20th, 24th and 27th of August 2019 and identification of the den in one of the study paddocks (Figure 1). A female wild dog was subsequently shot on the afternoon of 2 September 2019. No other sightings or signs of wild dogs were seen on the property until February 2020, well after the conclusion of this study. The identification, and subsequent removal, of the wild dog was not part of the original broader study; however, these events provided an excellent opportunity to study the behaviour of sheep in relation to a wild dog.

In total, 21 adult merino wethers and 47 adult merino ewes were monitored as part of the wider study. Animals were selected from larger flocks (approximately 200–300 head). Due to drought and destocking, animal availability was limited, and animals ranged in age from 1 to 8 years, although most animals (72%) were aged 1 or 2 years. Wethers and ewes were managed in separate paddocks under extensive commercial conditions, grazing a mix of native perennials, including buffelgrass (*Cenchrus ciliaris*), Flinders grass (*Iseilema* spp.) and Mitchell grass (*Astrebla* spp.).

Due to the opportunistic nature of the current study, only animals where devices recorded data for the full study period (7 August to 28 September 2019) were included in the current study. In total, 50 GPS tracking collars were included in the analysis (Figure 1).

### 2.2. Weather

Daily weather observations for the study period were obtained from the Long Paddock website [23]. Rainfall measurements were taken from on-property records; however, no rain was recorded during the current study period. Maximum daily temperature (T) in degrees Celsius and relative humidity (RH) at maximum daily temperature were used to calculate the temperature-humidity index (THI), or heat stress, experienced by animals during the study period. THI was calculated using the below Equation by Marai et al. [24] with THI:

THI = T − [(0.31 − 0.31 × (RH/100)) × (T − 14.4)](1)

### 2.3. GPS Tracking Collars and Data Analysis

Each sheep was fitted with a collar that contained an i-gotU GT-600 GPS logger (Mobile Action Technology Inc., Taipei, Taiwan) configured to collect a positional fix every 5 min. Raw GPS tracking data was downloaded using @TripPC (Mobile Action Technology Inc., Taipei, Taiwan) and analysed as per Fogarty et al. [25]. In short, erroneous locations were detected and removed; time, distance and speed between successive locations were calculated; speeds over 3 m/s were removed (as are commonly associated with GPS error) and movement metrics recalculated.

Due to the abnormal interference by farm staff with sheep on the day the wild dog was removed (2 September 2019), this day and all related data were removed from analysis. Overall, 52 days of data per GPS collar was analysed, comprising 26 days where a wild dog was present on the property (“wild dog present”) and 26 days directly after the wild dog was removed (“wild dog absent”).

Daily and hourly speed and distance metrics (total, mean, maximum and minimum) were calculated for each GPS device for each calendar day of the study, based on a 24-h period from midnight to midnight. In addition, basic behaviours (active/inactive) were calculated based on the speeds of each fixed interval. As such, speeds of ≤0.02 m/s were considered as inactive behaviours, i.e., standing/lying/resting, and speeds of >0.02 m/s were considered as active behaviours, i.e., grazing/walking [26,27,28]. Hourly and daily summaries were also calculated for each device whereby the total number of fixes recorded as active, per hour or day, was calculated and presented as a percentage of the total number of fixes recorded by that device for that hour or day.

### 2.4. Statistical Analysis

All statistical analyses were undertaken using R statistical software version 3.6.0 [29]. Linear mixed-effects models and subsequent *p*-values were developed using the ‘lme4′ [30] and ‘lmerTest’ [31] packages with significance determined as *p* ≤ 0.05. The data analysis focused on using mixed-effects models to understand the effect of dog presence on the daily distance travelled by animals.

A couple of key points of the statistical analysis: (1) the effect of dog presence was used in the model as a fixed effect factor with two levels: dog, no-dog; (2) a fixed effect factor with two levels was used in the model to account for systematic differences between paddocks—it is important to note that the two paddocks house different animal categories and the experiment only had two paddocks with one paddock per animal category; and (3) the analysis utilised a linear mixed-effects model with fixed effects of THI at maximum daily temperature (maximum THI), time spent active, dog presence and paddock and a random effect of animal to account for the repeated observations on the same animal.

The residuals versus predicted values plot suggested considerable heteroscedasticity; therefore, a natural logarithmic transformation was used on the response variable (daily distance travelled). Results are presented for the model with the response variable as the natural logarithm of the daily distance travelled.

## 3. Results

### 3.1. Daily Distance Travelled and Activity

**Figure 2:**
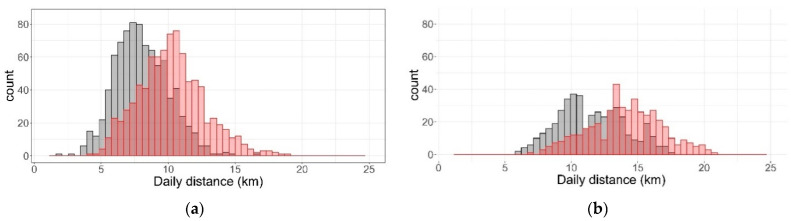
Frequency distribution of daily distance travelled for (**a**) ewes and (**b**) wethers when a wild dog was present (red) and absent (black).

During the period where a wild dog was present, sheep travelled on average, 11.6 ± 3.12 km/day with a maximum distance travelled of 21 km/day (Table 1) and wethers travelled, in general, further each day than ewes (Figure 2). During the period where a wild dog was present, there were 162 occurrences (17 animals over 22 days) where wethers travelled > 15 km/day and 35 occurrences (18 animals over 12 days) where ewes travelled > 15 km/day. Overall, sheep spent 69.3% of the day being active during this period and visited water approximately 1.1 (median = 1) times per day (Table 1).

After the wild dog was removed, sheep travelled, on average, 9.2 ± 2.73 km/day with a maximum distance of 17.6 km/day. This is a 20% reduction in the daily distance travelled by animals between these two periods (wild dog present and wild dog absent). Again, wethers travelled further than the ewes during this period (Figure 2); however, the number of occurrences where animals travelled distances > 15 km/day were reduced for both groups. For wethers, there were 49 occurrences (17 animals over 10 days), while just one occurrence of an animal travelling > 15 km/day was recorded for the ewes. This was a reduction in the number of occurrences animals travelled > 15 km/day of 69.8% and 98% for wethers and ewes, respectively. After the dog was removed, sheep were, overall, active for approximately 68.7% of the day and visited water 0.9 (median = 1) times per day (Table 1).

Initial analyses also indicated a negative relationship between THI at maximum daily temperature and daily distance travelled. However, the results from Table 1 indicate that the difference in summary statistics for maximum and minimum THI was approximately only 3.3 points for the periods when the wild dog was present and absent on the farm.

### 3.2. Diurnal Activity

**Figure 3:**
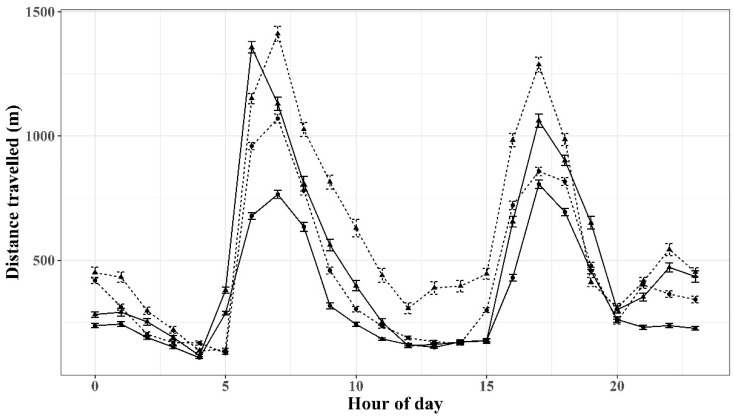
Average distance travelled per hour of day for ewe and wether groups. Averages presented for both when a wild dog was present and absent. Ewes (●); wethers (▲); dog present (dashed line); dog absent (solid line).

Two distinct periods of activity in terms of distance travelled per hour were observed from approximately 0600 to 0900 h and again from 1600 to 1900 h for both the wether and ewe flocks. These active periods were observed both during the time the dog was present and after the wild dog was removed (Figure 3). An additional smaller peak of activity was also observed later in the evening, except for the ewes after the wild dog was removed. During periods of active behaviour, wethers appeared to be more active than ewes with larger distances travelled per hour recorded.

During daylight hours, the wethers were significantly more active between 0600 and 1700 h when the wild dog was present compared to after it was removed. The ewes, however, were significantly more active between 0600 and 1100 and then again from 1500 to 1600 h, when the wild dog was present. Evening activity was much less than daylight hours for both ewes and wethers, although higher levels of activity were seen when the dog was present between the hours of 2100 and 0000, and 2100 and 0200 h for ewes and wethers, respectively.

### 3.3. Drivers of Daily Distance Travelled

Differences in paddocks, behaviour, weather (THI) and presence/absence of the wild dog on the farm were modelled against daily distance travelled to understand the variables that influenced the daily distance travelled by sheep. The model fit suggests that THI at maximum daily temperature, number of daily water visits, time spent active, paddock and wild dog presence were all associated with the distance travelled by sheep (Table 2 and Table 3). Total daily distance travelled reduced with an increase in THI. Inversely, the number of times animals visited water each day, paddock, time spent active and the presence of a wild dog all increased distance travelled.

Parameter estimates and standard errors for daily distance travelled are given in Table 2. Because the model was fitted with distance travelled transformed by natural logarithm, the parameters have a multiplicative interpretation. For example, the parameter associated with paddock (wethers) was estimated to be 0.196 (Table 2). Therefore, the influence of paddock is associated with a $100\times \left(e^{0.196}-1\right)=$ 21.65 (±3.46) percent change in total daily distance travelled. Using this same approach, results suggest that a unit increase in maximum THI is associated with a −2.17 (±0.10) percent change in distance travelled. In contrast, a unit increase in the time spent active, daily water visits and the presence of the wild dog were associated with a 2.02 (±0.10), 7.90 (±0.50) and 12.63 (±0.90) percent change in distance travelled, respectively. Similar results were seen when flocks (ewes and wethers) were split and analysed separately (Table 3), although the effect of the wild dog on the wether flock was greater (14.57 (±1.51) percent change) than that for the ewe flock (11.74 (±1.21) percent change).

## 4. Discussion

Prey species, such as sheep, have evolved a range of anti-predator responses to both predator encounters and generalised threatening stimuli, such as loud noises or sudden events [32]. For sheep, anti-predator responses can be broadly divided into two types; those they display due to the immediate threat or presence of a predator [33], such as foot stamping, vocalisation and fleeing [34]; and those which have evolved to help reduce the likelihood of detection and capture by a predator, i.e., flocking [33]. When exposed to a threat, the most common response of sheep is to flee, and sheep flocks have been reported to respond earlier to a threat than solitary animals [35]. This is likely due to increased vigilance as a result of an increase in animal numbers [36] and the strong flocking and synchronisation instinct that sheep possess [37,38] as a form of protection. Due to this innate flock-based movement of sheep and the flock-related behaviours identified in previous research [22], the current study focused on identifying changes in the behaviour of the flock when a wild dog was present on the property, rather than changes of individual animals. The intent was to determine if the presence of a predator, such as a wild dog, impacted the behaviour of rangeland sheep in a way that could be detected by on-animal sensors for use as a future early alert and warning of wild dog presence.

Results indicated that for both wethers and ewes, the daily time spent active, maximum THI, number of water visits and presence or absence of the wild dog significantly impacted distance travelled. Although the change in maximum THI across the study period was not to the extremes experienced throughout a full year in Western Queensland, it was enough to be identified as a factor contributing to a decrease in daily distance travelled. These findings are similar to those of Thomas et al. [39], who reported that sheep had a lower horizontal travel velocity and did not travel as far from water on warmer days. Likewise, previous studies have linked heat stress in sheep with a decrease in feed intake [40] and an increase in time spent lying/sleeping [41]. Water visitation has also been reported to affect the distance travelled, with an increase in distance travelled associated with an increase in watering events from one to two per day [42]. Despite these factors having a significant effect on the daily distance travelled by sheep in this study, this effect was not as strong as that for the presence or absence of a wild dog.

In wildlife species, it has been shown that the presence of predators can influence the behaviour of prey species by changing the location or times at which prey forage [43], while the fear associated with chronic perceived predation risk can lead to changes in foraging activity [44]. In the current study, the diurnal patterns of the two flocks indicated that animals were active/inactive during similar time periods despite the presence or absence of a wild dog (Figure 2). However, animals were more active throughout much of the day when the wild dog was present. In particular, the wethers were significantly more active than the ewes between 0700 and 1700 h. This difference in activity in the wether flock could be due to the wild dog being more active around these animals as many wild dog sightings (fresh tracks/scats/signs of a wild dog) were observed in or around this paddock. In addition, the den of the wild dog was located on the eastern side of the paddock housing the wethers (Figure 1). As such, it is plausible that the wethers interacted more frequently with the wild dog and could explain why, overall, the wethers were more active and travelled further than the ewes. However, the clear differences in active periods for both the wethers and ewes suggest that dog presence may be detectable from changes in movement data that is restricted to expected periods of active or inactive behaviour, although further work is needed.

The differences in activity patterns of the sheep in this study begin to quantify the secondary impacts wild dogs can have on sheep in a rangeland grazing environment beyond the specific injury and death of individual animals. Generally, the impact of dog predation is calculated in terms of stock losses [8] or the psychological impacts to the producer [13]. However, the increase in distance travelled by animals in this study suggests that the presence of a predator may have a negative effect on the energy expenditure of animals and thus a negative impact on productivity [45]. In wild ungulate species, previous reports have shown that predator presence can influence habitat usage [46], diurnal patterns [47] and time spent vigilant or grazing [48,49]. While the specific impact predator presence has on sheep productivity will be difficult to empirically study, it does warrant further investigation due to the potential negative effects predator presence has on productivity.

Being able to identify changes in the behaviour of livestock autonomously and in close to real-time could assist in alerting producers to health and welfare issues on-farm while also facilitating changes to livestock management, monitoring and intervention. Thus far the use of on-animal sensors in sheep have been shown to identify lameness [50], parturition [25,51,52], oestrus [53], paddock utilisation [26] and worm burden [54]. Unfortunately, the 5 min sample interval of GPS tracking data used in this study did not allow for frequent enough data to capture true moments of harassment, such as a sudden increase in speed or centripetal rotation by the flock, as reported previously [22]. Likewise, direct interactions of the wild dog and the sheep fitted with livestock tracking collars were not observed. However, the significant increase in daily distance travelled of sheep during the time a wild dog was present on-farm, and the changes to diurnal activity that there are detectable differences in the behaviour of sheep flocks when in the presence of a wild dog.

These results highlight the potential for on-animal sensors to alert producers to the presence of a wild dog on their property, enabling producers to make management changes to affected flocks and/or intervention such as targeted control measures for wild dogs. This ability to remotely detect predation issues on-farm in the future has enormous benefits to production, profitability, animal welfare and producer wellbeing. An acknowledged limitation of the current case study is that it was undertaken on a single property in Western Queensland and, as such, it is unknown if the behaviour of the sheep and wild dog under these conditions is typical of all rangeland sheep in Australia. As such, additional work needs to be undertaken to identify the behavioural changes of rangeland sheep in response to the presence of a wild dog and to obtain baseline information on the typical daily behaviour of sheep. Further research should also explore how early warning systems for dog presence might be used by producers to improve control measures for this pest. Further to this, the economic benefits of implementing on-animal sensors as part of a wild dog program will also need to be understood if these systems are to bring genuine benefits to the industry.

## 5. Conclusions

The results of this study suggest that the presence of a wild dog influences the daily behaviour of sheep in a rangeland environment by increasing the distance travelled. Further work needs to be undertaken to identify the changes exhibited by sheep in response to a wild dog in alternate environments, as well as the behavioural and spatial changes that occur when sheep flocks directly interact with a wild dog. However, these results highlight the potential for GPS tracking collars and on-animal sensors to detect behavioural changes of sheep in the presence of a wild dog, and the potential for alerting producers earlier to the presence of wild dogs, thus allowing for improvements to management practices and interventions to be implemented.

## Figures and Tables

**Table 1:** Summary statistics for variables across periods where a wild dog was present and absent.

Variable	Dog Present					Dog Absent				
	Min	Median	Mean	Max	S.D.	Min	Median	Mean	Max	S.D.
Daily distance (km)	4.2	11.1	11.6	21.0	3.12	1.9	8.9	9.2	17.6	2.73
Percent Active (%/day)	45.3	69.9	69.3	89.5	6.25	39.5	69.3	68.7	85.1	6.35
Maximum THI	18.4	23.4	23.4	27.3	2.23	21.2	27.7	26.7	30.6	2.57
Minimum THI	4.0	10.8	10.2	16.2	3.12	6.2	13.7	13.4	19.0	3.87
Water Visits	0	1	1.1	4	0.73	0	1	0.9	4	0.83

S.D. = standard deviation.

**Table 2:** Parameter estimates for daily distance travelled (km)—overall.

Parameter	Estimate	S.E.	*p*-Value
Intercept	1.359	0.068	0.041
Slope for maximum THI	−0.026	0.001	<0.001
Slope for dog presence	0.119	0.009	<0.001
Slope for paddock (wethers)	0.196	0.034	<0.001
Slope for water visits	0.076	0.005	<0.001
Slope for time spent active	0.020	0.001	<0.001

Variance components estimates: Animal = 0.012, Error = 0.033. S.E. = standard error.

**Table 3:** Parameter estimates for daily distance travelled (km) for wethers and ewes separately.

	Wethers			Ewes		
Parameter	Estimate	S.E.	*p*-Value	Estimate	S.E.	*p*-Value
Intercept	1.583	0.124	<0.001	1.375	0.083	0.013
Slope for THI max	−0.022	0.002	<0.001	−0.029	0.002	<0.001
Slope for dog presence	0.136	0.015	<0.001	0.111	0.012	<0.001
Slope for water visits	0.081	0.009	<0.001	0.078	0.006	<0.001
Slope for active	0.018	0.001	<0.001	0.021	0.001	<0.001

Variance components estimates: Wethers—Animal = 0.003, Error = 0.030; Ewes—Animal = 0.016, Error = 0.034. S.E. = standard error.

## Data Availability

The datasets presented in the current study are not publicly available due to privacy restrictions but may be made available from the corresponding author upon reasonable request.
